# Exploring conformational preferences of proteins: ionic liquid effects on the energy landscape of avidin†

**DOI:** 10.1039/d0sc04991c

**Published:** 2020-10-23

**Authors:** Talia A. Shmool, Laura K. Martin, Coby J. Clarke, Liem Bui-Le, Karen M. Polizzi, Jason P. Hallett

**Affiliations:** Department of Chemical Engineering, Imperial College London London SW7 2AZ UK j.hallett@imperial.ac.uk +44 (0)20 7594 5388; Imperial College Centre for Synthetic Biology, Imperial College London London SW7 2AZ UK

## Abstract

In this work we experimentally investigate solvent and temperature induced conformational transitions of proteins and examine the role of ion–protein interactions in determining the conformational preferences of avidin, a homotetrameric glycoprotein, in choline-based ionic liquid (IL) solutions. Avidin was modified by surface cationisation and the addition of anionic surfactants, and the structural, thermal, and conformational stabilities of native and modified avidin were examined using dynamic light scattering, differential scanning calorimetry, and thermogravimetric analysis experiments. The protein-surfactant nanoconjugates showed higher thermostability behaviour compared to unmodified avidin, demonstrating distinct conformational ensembles. Small-angle X-ray scattering data showed that with increasing IL concentration, avidin became more compact, interpreted in the context of molecular confinement. To experimentally determine the detailed effects of IL on the energy landscape of avidin, differential scanning fluorimetry and variable temperature circular dichroism spectroscopy were performed. We show that different IL solutions can influence avidin conformation and thermal stability, and we provide insight into the effects of ILs on the folding pathways and thermodynamics of proteins. To further study the effects of ILs on avidin binding and correlate thermostability with conformational heterogeneity, we conducted a binding study. We found the ILs examined inhibited ligand binding in native avidin while enhancing binding in the modified protein, indicating ILs can influence the conformational stability of the distinct proteins differently. Significantly, this work presents a systematic strategy to explore protein conformational space and experimentally detect and characterise ‘invisible’ rare conformations using ILs.

## Abstract 2

Revealing solvent and temperature induced conformational transitions of proteins and the role of ion–protein interactions in determining the conformational preferences of avidin in ionic liquids.

## Graphical abstract

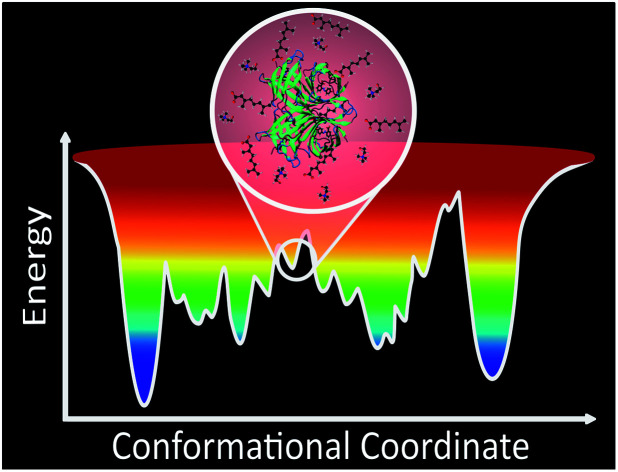

## Introduction

Proteins are inherently dynamic and can transiently sample an ensemble of conformational states.^1^ The heterogeneity of the conformational ensemble (for example, compact globular states, localised secondary structure, and coil-like states) reflects the broad distribution of conformers, the different shapes and stabilities, a protein can adopt in its immediate environment.^1,2^ The energy landscape of a protein encodes the relative stabilities of its different conformational states and the energy barriers that separate the conformations.^2^ The populations of the conformations follow statistical thermodynamic distributions, and the heights of the energy barriers separating the conformations define the timescale of conformational transitions.^3–5^ We can visualise that for a ligand-binding protein in solution, if the free energy barriers are low relative to the Boltzmann energy (*k*_B_*T*), thermal fluctuations can lead to significant populations of multiple conformers.^2,6^ A ligand can then interact with the lowest energy conformer, or with one of a number of higher energy conformers that are populated in solution. Upon a population shift, the relative populations of conformers that already pre-exist in solution redistribute.^1^ However, experimental techniques are limited when determining conformational diversity, particularly since structural characterisation of conformations, other than the most prevalent one, in solution is intensely challenging.^4,7–9^ Despite this, studying protein conformational diversity, key for understanding protein function, continues to be extensively investigated using a wide range of theoretical models from protein physics to Darwinian selection.^2,10–12^ The goal to determine which selective pressures can induce a stable protein conformation is further complicated by the large conformational space a protein can sample, preferential interactions available, and environmental conditions.^13^ This begs the question: Can we tune environmental factors to select for more stable protein conformations?

Avidin, a homotetrameric glycoprotein naturally occurring in egg whites, is an ideal model system for studying protein susceptibility to stress-induced conformational changes, due to its high structural and thermal stability to extremely harsh environmental conditions.^14–20^ The overall fold of each monomer in avidin is a classical β-barrel formed by eight antiparallel β-strands.^17^ The ligand binding site is a pocket, located at the ends of each β-barrel which contains both polar and aromatic residues.^18,19^ Avidin is composed of 72 basic residues and 48 acidic residues per molecule, resulting in an isoelectric point of 10.5, below which avidin has a net positive charge.^20^ At a pH below 4.5, the net positive charge on avidin increases as the glutamate, and then the aspartate, residues become protonated.^16^ Above a pH of 10.5, the lysine residues are deprotonated, resulting in a net negative charge on avidin.^16,20^ When biotin is bound to avidin the interaction promotes remarkably high stability to the avidin–biotin complex (*K*_d_ ∼ 10^−15^),^14,15^ and the high pI and carbohydrate content of avidin allows it to bind non-specifically to ligands and molecules other than biotin. Additionally, the ability to chemically modify the surface of avidin has been exploited for a wide range of biochemical, biophysical, and medical applications, as well as in computational studies examining the conformational changes of avidin induced by ligand binding.^14^ It can be envisioned, that the native unbound state of avidin exists as a dynamic ensemble of rapidly interconverting conformations, which can be described by a relatively rugged energy landscape with many local energy minima and energy barriers.^4,5^ A biochemical modification of avidin would change the relative free energies of individual conformations as well as the energy differences between conformations. Thus, we chose to use avidin to investigate the conformational transitions, stability against thermal denaturation, and conformational preferences of proteins under specific environmental conditions.

In recent years, room-temperature ionic liquids (ILs) have been explored as potential solvents, providing unique environments for proteins, enhancing solubility, stability, and catalytic activity of proteins and enzymes.^21^ Given the wide range of ions available, by choosing specific ion combinations, ILs can be tailored to give unique properties (for example, immiscibility, low nucleophilicity, acidity, and solvation strength), and alter protein conformations in a tuneable sense.^21,22^ For example, protic ILs have been shown to stabilise the secondary structure of proteins above denaturing temperatures, while aprotic ILs can have destabilising effects.^22,23^ Additionally, biocompatible ILs, for example choline-based ILs, can improve the long-term stability and solubility of proteins, although electrostatic binding of nucleophilic anions with protein surfaces can disrupt internal packing interactions, crucial for the structural stability of proteins.^1,22^ Since ionic interactions play a major role in understanding the physicochemical and biological phenomena involved in protein folding and unfolding processes, a great deal of work has focused on studying the versatile properties of ILs, and the interactions responsible for stabilising and destabilising proteins.^21,22,24^ Yet, to date, no experimental study has investigated the role of ion–protein interactions in determining the conformational preferences of proteins in ILs.

Inspired by the high tunability of ILs, for the first time, we aimed to determine whether we can use ILs to stabilise and experimentally find specific, rarely populated protein conformations from a heterogeneous conformational ensemble, where environmental factors result in the interplay of different conformations. This would provide us with a unique opportunity to experimentally determine the conformational energy landscape of proteins, and find ‘invisible’ conformers, accessible by conformational transitions from the native state. We chose seven biocompatible IL and buffer solutions (of a comparable pH range to the IL solutions), to investigate and uncover the conformational space of the robust and reliable avidin system and determine the favoured conformations under particular environmental conditions. Choline amino acid and fatty acid ILs were chosen as these are widely used and well characterised in the literature, with toxicities much lower than traditional ILs, such as imidazolium-based ILs.^21,22^ Additionally, amino acids are particularly attractive as potential anions, due to their inherent biocompatibility, and since the presence of additional hydroxyl, carboxylic acid and amide groups on the side chain have been shown to improve the thermal stability of proteins.^22^ We also note that to our knowledge, fatty-acid-based ILs have yet to be examined for improving the conformational stability of proteins, and thus are of particular interest to us. The IL solutions synthesised include choline phenylalaninate [Cho][Phe], choline methioninate [Cho][Met], choline aspartate [Cho][Asp], choline geranate [Cho][Ger] and choline hexanoate [Cho][Hex], as well as aqueous solutions of the salts choline chloride [Cho]Cl and choline dihydrogen phosphate [Cho][DHP], specifically chosen to display a range of different functionalities and interactions between anions and protein. Geranate ([Ger]) and hexanoate ([Hex]) are medium chain fatty acids, containing a significant hydrophobic component that can interact with the hydrophobic patches of avidin and its binding site.^18^ Phenylalanate ([Phe]), aspartate ([Asp)], and methionate ([Met]) are aromatic, acidic and thioether containing amino acids, respectively, introducing specific hydrogen bonding interactions between avidin and the IL, resulting in different avidin conformers. Finally, dihydrogen phosphate ([DHP]) and chloride (Cl) are common inorganic counterions for ILs and have been shown to increase protein stability *via* hydrogen bonding and electrostatic interactions,^25^ making them valuable for studying molecular confinement effects on avidin stability.

We took advantage of our ability to uniquely modify avidin and study the effects of ILs on the conformations, stability, thermostability, and binding behaviour of unmodified and modified avidin using a multi-technique approach. First, the conformational and thermal stability between unmodified and modified avidin were characterised and differences in the conformational states were determined using dynamic light scattering (DLS), differential scanning calorimetry (DSC), and thermogravimetric analysis (TGA) experiments. Then, we examined the effects of different ILs at varying concentrations on the structure and thermal stability of the proteins using small angle X-ray scattering (SAXS), differential scanning fluorimetry (DSF) experiments, thermal shift assays (TSA), thermodynamic data analysis, and variable temperature circular dichroism (CD) spectroscopy experiments. Finally, in order to further study the effects of ILs on avidin binding and correlate thermostability with conformational heterogeneity and preferences we conducted a binding study. This novel systematic approach enables us to probe the conformational landscape of the distinct protein systems and build a comprehensive picture of the heterogeneity of avidin in the presence of different ILs.

## Results and discussion

In order to characterize the conformational ensembles of unmodified and modified avidin and assess the thermal behaviour of the different avidin conformers, the hydrodynamic radius (*R*_h_), decomposition temperature and any reversible phase transitions were measured using DLS, TGA and DSC, respectively. The hydrodynamic radius (*R*_h_), the effective radius of an ion, including the bound solvent molecules on its surface, was measured by DLS for aqueous native avidin as 26.4 ± 0.3 Å, in reasonable agreement with the literature (Table 1).^26^ Upon cationisation, a slight increase in *R*_h_ (27.9 ± 0.2 Å) was observed, which then increased significantly once the surfactants were added, with aqueous modified avidin *R*_h_ measuring 49.5 ± 0.4 Å. Freeze-dried modified and unmodified avidin were also characterised by DSC and TGA (see ESI Fig. S1†). A melting point for modified avidin was observed at 23.7 °C, in reasonable agreement with the literature for this modification procedure on other proteins,^27^ while for unmodified avidin no phase transitions were detected in the range of −80 to 100 °C (Table 1 and ESI Fig. S1†). By TGA, the decomposition temperature observed for modified avidin (381 °C) was much higher than that of unmodified avidin (263 °C). This provides evidence that there is a difference in the conformational ensemble of unmodified and modified avidin, respectively, shown by the higher degradation temperature and the presence of a melting transition observed for the conformational states of modified avidin.

**Table:** Calculated hydrodynamic radius (*R*_h_), decomposition temperature and melting temperature for the unmodified, cationised and modified avidin. N/A indicates no decomposition/melting data available, — indicates no transition observed

Avidin sample	Hydrodynamic radius (*R*_h_) (Å)	Decomposition temperature (°C)	Melting temperature (°C)
Unmodified	26.4 ± 0.3	263	—
Cationised	27.9 ± 0.2	N/A	N/A
Modified	49.5 ± 0.4	381	23.7

Previously, such modifications have been shown to cause changes in the relative prevalence of β-sheet, α-helix and unordered secondary structures,^28^ specifically an increase in α-helix and decrease in β-sheet content. We interpret the influence of modification as a shift from β-strand to α-helical conformers of avidin, attributed to the binding of surfactant to avidin, which could alter the conformational flexibility of the exposed loops connecting the β-strands of avidin, and thus the accessibility of avidin to aqueous solvent.^29,30^ Given that disordered proteins have been shown to be more heat stable and soluble compared to their folded counterparts,^2^ it is not surprising that modified avidin exhibits a raised decomposition temperature. Thus, it can be suggested that the process of modification altered the conformational ensemble of avidin to favour a thermally stable, rarely populated conformation.

**Fig. 1:**
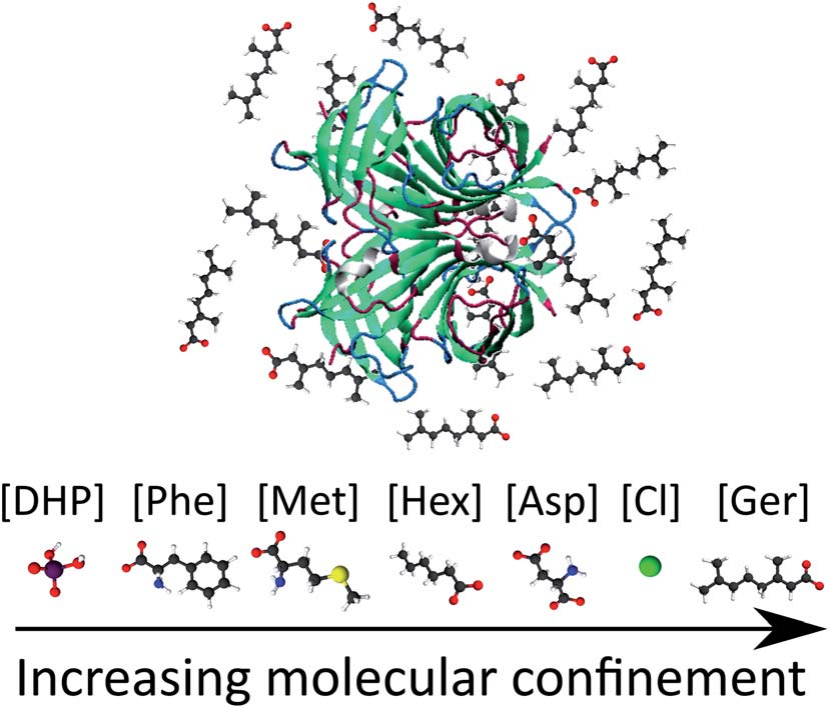
Schematic of molecular confinement of avidin by [Cho][Ger]. The increasing effect of molecular confinement of avidin by the IL, *via* ion–protein interactions, is shown for the anions of the different ILs examined.

Having established that our experiments are starting from different conformational ensembles, we further examined the structure of the unmodified and modified avidin conformers and the effects of IL composition and concentration on changes in the compaction of avidin. By performing SAXS experiments (see ESI Fig. S2†) we found that unmodified avidin in IL and in aqueous buffers are comparable in secondary structural compactness, as indicated by the radius of gyration (*R*_g_) values (Table 2). Most notably, at higher IL concentrations, *R*_g_ was reduced and the degree of compaction of avidin increased. It can be suggested that with an increase in IL concentration, the coordination of ions to the α-helices and β-sheets increased proportionally, reducing the number of exposed charged surfaces, and compacting local protein folds (Fig. 1).^29,30^ This is consistent for all of the ILs examined, apart from [Cho][Hex], which showed a strong background signal in the SAXS measurement at 50 wt%, attributed to a combination of the high hydrophobicity of [Cho][Hex] relative to the other ILs and the high IL content used, as previously observed in aqueous solution of the IL choline iso-butyrate.^31^ Furthermore, despite the high, potentially denaturing pH of the [Cho][Phe] and [Cho][Met] environments, the *R*_g_ value of unmodified avidin decreased (albeit higher initial *R*_g_ values) with increasing concentration. This consistent trend could suggest a conformational shift from conformer of higher conformational flexibility to compact conformers, and preferences that restrict the conformational search by chain compaction.^4,32^

Radius of gyration (*R*_g_) values calculated for a range of IL and buffer solutions from SAXS data (see ESI Fig. S2 for raw data plots and fits). The pH and concentration of each solution is reported

**Table:** 

Buffer solution	Concentration (M)	pH	Unmodified protein *R*_g_ (Å)	Modified protein *R*_g_ (Å)	Surfactant micelles *R*_g_ (Å)
Citrate–phosphate buffer	0.1	5.1	28.3	47.8	15.3
Phosphate buffer	0.1	7.1	28.4	48.5	15.9
Tris buffer	0.1	9.1	28.9	48.5	16.3

**Table:** 

IL	Concentration (wt% IL)	pH	Unmodified protein *R*_g_ (Å)	Modified protein *R*_g_ (Å)	Surfactant micelle *R*_g_ (Å)
[Cho][Asp]	10	7.36	29.0	44.1	15.7
	50	7.85	28.0	—	16.6
[Cho][Ger]	10	8.23	28.6	50.0	—
	30	9.37	27.1	49.9	—
[Cho][Hex]	10	8.60	28.1	47.1	15.6
	50	10.07	28.4	—	—
[Cho][Phe]	10	10.69	29.6	44.4	15.0
	50	11.11	29.4	—	17.1
[Cho][Met]	10	10.85	28.4	46.3	16.0
	50	11.45	28.2	—	18.9
[Cho]Cl	10	4.66	28.2	47.0	17.3
	50	3.75	27.4	—	16.1
[Cho][DHP]	10	—	30.1	—	16.4

For the modified avidin, two distinct structures were observed in the SAXS data with different radii. By control experiments with surfactant-only solutions (no avidin present, see ESI Fig. S3†) and comparison to the DLS data for modified avidin, it was established that the larger structures (*R*_g_ ∼ 45–50 Å) were modified avidin, while the smaller structures (*R*_g_ ∼ 15–19 Å) were free surfactant micelles, formed by excess surfactant dissociating from the modified structure under aqueous conditions.^33^ Since the hydrophilic head of the surfactant molecules can interact with the ions in solution and form micelles, the effect of molecular confinement and proportion of ions available that could compact the avidin structure likely changed.^34^

Table 2 shows that for the modified avidin at 10 wt% IL, almost all samples, except in [Cho][Ger], exhibited reduced *R*_g_ and increased compactness, similar to the compaction effect observed for unmodified avidin. Specifically, preferential hydrophobic interaction between the long hydrocarbon tail of the geranate anions and the hydrocarbon component of the surfactant molecules, could result in reduced interactions between the IL and modified avidin, partly explaining the lower observed compaction in [Cho][Ger]. At 50 wt% IL, features representing the modified protein structures were poorly defined and could not be fitted (see ESI Fig. S4†), while the micelle peaks became more defined. The likely conclusion is that the ions of the ILs were in competition with the surfactant molecules, potentially resulting in ion exchange within the nanoconjugates. This disrupted the nanoconjugates sufficiently that they were no longer detected by SAXS and limited the effective protective effects of the ions on the hydrophobic surfaces of the protein. While the conformational sampling of modified avidin was affected,^6,34^ it is clear that the avidin could be driven toward the compact conformations by ILs. Significantly, in order to understand the conformational preference of avidin, it is essential to obtain information about the structure and stability behaviour of the different conformers in highly-defined environments.

Next, we aimed to further study the conformational heterogeneity of avidin and connect the observed macromolecular confinement with thermal stability behaviour of the different conformers generated. Control differential scanning fluorimetry (DSF) experiments revealed that avidin was most stable at pH 7.1 (0.05 M citrate/phosphate buffer), with *T*_m_ of 88.7 °C. At pH 5.1 (0.05 M citrate/phosphate buffer), the *T*_m_ was slightly reduced (86.4 °C), alluding to the contribution of pH, linked to net surface charge and the stability of avidin in solution.^35^ Similarly, at pH 9.1 (0.05 M tris buffer), the *T*_m_ was further reduced (82.9 °C). Accounting for differences in conditions and method used, measured *T*_m_ values were in reasonable agreement with the literature.^36^ The behaviour of avidin in alkaline pH (in the range pH 7.1–9.1), is in agreement with the effects of molecular confinement of avidin by the solvent molecules, *via* solvent–protein interactions, leading to conformational redistribution and a population shift toward a thermally less stable avidin conformer.^4,29,32^

*T*_m_ values were then measured in aqueous IL solutions at 10 wt%. We found that [Cho][Asp] increased avidin thermostability by the greatest amount, with *T*_m_ exceeding that of the pH 7.1 control by 4.5 °C. In 10 wt% [Cho]Cl solution, with pH 4.66, the *T*_m_ was 87.6 °C, above that of the pH 5.1 control. Most notably, [Cho][Met] and [Cho][Phe], both with pH > 10.7, gave *T*_m_ of 85.8 °C and 86.6 °C, respectively. These values were relatively higher than the pH 9.1 control (82.9 °C), emphasising that the presence of IL can mediate, and in some cases enhance the thermostability of avidin under specific conditions.

To further examine the idea that molecular confinement by the IL influences the thermal stability and conformations of avidin, ‘buffered’ samples of the IL solutions (10 wt% of [Cho][Asp], [Cho][Met], [Cho][Phe] and [Cho]Cl) containing citrate/phosphate buffer (0.05 M) were also tested. Given that the buffered solutions contained a larger number of components, the effect of solvent-induced confinement on the conformation and stability of avidin were expected to be larger. In all cases the pH was brought closer to neutral and an increase in *T*_m_ was seen. In [Cho][Asp], which was already near neutral, pH decreased from 7.4 to 7.1, but only a +0.1 °C increase in *T*_m_ was observed. For [Cho][Met] and [Cho][Phe], adding buffer decreased the pH similarly in each case (from 10.9 to 10.4 and 10.7 to 10.3, respectively) and the *T*_m_ values increased by +2.3 °C and +0.9 °C, respectively. Unsurprisingly, the increase in *T*_m_ (+2.8 °C) was larger in [Cho]Cl, where the pH change was much greater, increasing from 4.7 to 6.9. The observation that adding buffer can increase the *T*_m_ even when the pH change is small, as exemplified in the case of [Cho][Met], indicated more favourable conditions for thermostability in this complex mixture. It can be suggested that in buffered solutions, compared to neat IL, a different number of solvent molecules can associate on the avidin surface, and form larger patches, increasing the conformational stability of avidin and the *T*_m_, as we observe in our work.^37,38^

**Fig. 2:**
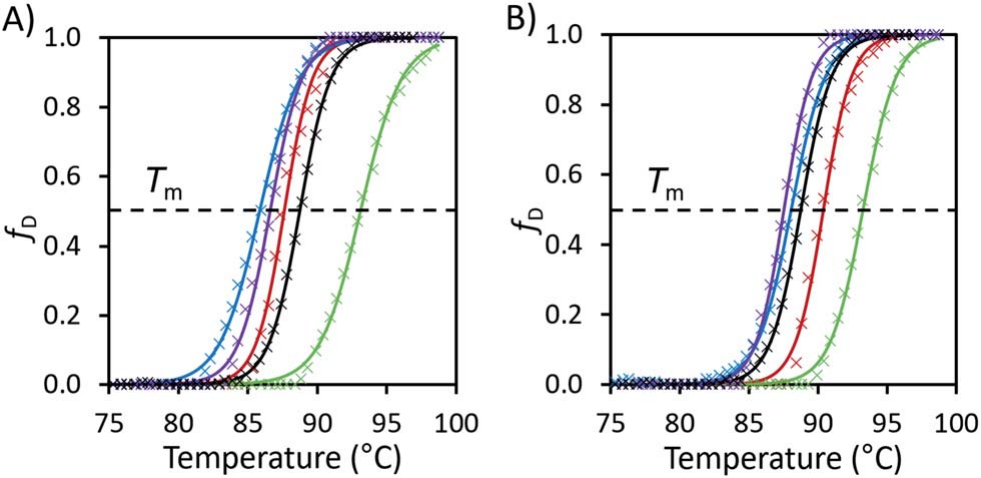
(A) Fraction denatured (*f*_D_) of unmodified avidin against temperature in 10 wt% solutions of [Cho][Asp] (green), [Cho][Met] (blue), [Cho][Phe] (purple), [Cho]Cl (red) and pH 7.1 citrate–phosphate buffer (black) with raw data (×) and fitted Boltzmann distribution curves (smooth lines). (B) The fraction denatured plots of the same IL solutions with additional 0.05 M, pH 7.1 citrate–phosphate buffer. Plots calculated from the raw fluorescence DSF data (see ESI Fig. S5 and S6 for raw data, Table S1 for thermodynamic properties and Method S1 for calculation methods†).

We illustrate the barrier to denaturation for avidin in the different ILs with plots of the fraction of the protein denatured against temperature as determined from the DSF data (Fig. 2, see ESI Fig. S5 and S6†), with *T*_m_ representing the temperature at which half the protein is denatured. Advantageously, this method highlights not only the value of *T*_m_ in each case, but also the temperature range over which denaturation occurs and approximate the rate of denaturation.^27^ Using the DSF data we were able to calculate the thermodynamic properties of the denaturation process (ESI Method S1 and Table S1†). The thermodynamic data revealed a key trend: the enthalpy and entropy change (Δ*H*_m_ and Δ*S*_m_, respectively) of avidin denaturation were both higher when citrate–phosphate buffer was added to the aqueous IL solution. This effect was most prominent in [Cho][Met]; in the aqueous IL solution (in the absence of buffer) Δ*H*_m_ increased from 161 kJ mol^−1^ to 205 kJ mol^−1^ upon the addition of buffer to the solution, and Δ*S*_m_ also increased from 1880 J K^−1^ mol^−1^ to 2330 J K^−1^ mol^−1^. As discussed earlier, the addition of buffer also resulted in an increase in *T*_m_ for avidin in all IL solutions, indicating that despite the increase in entropic driving force for denaturation in this more complex, higher energy system, the balance between enthalpy and entropy of denaturation overall resulted in a higher thermal barrier to denaturation. These findings support the concept of molecular confinement, involving the strong, direct associations between the molecules of the buffered ILs and charged or polar groups of avidin. Specifically, for the unbuffered IL solutions, where the effects of molecular confinement were less pronounced, enhanced conformational motions enabled avidin to jump out of wells and explore different conformational environments. However, in the case of the buffered IL solutions, a larger number of molecules were in contact with avidin compared to in aqueous IL solutions. Consequently, in buffered ILs the greater molecular confinement imparted resulted in reduced flexibility and avidin sampled a sufficiently low energy conformation, so stable that it became trapped in an energy minimum that exceeded the available thermal energy. We highlight that in [Cho]Cl the increase in enthalpy and entropy change was the smallest (Δ*H*_m_ from 229 kJ mol^−1^ to 246 kJ mol^−1^ and Δ*S*_m_ from 2620 J K^−1^ mol^−1^ to 2730 J K^−1^ mol^−1^); however, the addition of buffer provided the greatest increase in thermal stability in this IL. This suggests that in this system the local conformations of avidin became trapped in the buffered IL by energy minima that were relatively more stable than *kT*. Thus, it appears that confinement is strongly dependent on the solvent used and is linked to conformational states. We note that while the results obtained for avidin in buffered IL imply that increased molecular confinement can enhance conformational stability,^38^ additional work should be conducted to assess the effect of internal structure in increasing thermal stability.

Notably, DSF could not be used to reliably extract information about protein stability for modified avidin or in [Cho][Hex] or [Cho][Ger] due to high background fluorescence caused by hydrophobic interactions between the SYPRO orange dye and the hydrocarbon chains of the IL anions. Nonetheless, the DSF data demonstrated that the aqueous environment was important for the thermostability of the avidin conformers, and distinct conformers with different thermostabilities were present in each IL solution.

Thus far we have provided extensive evidence that the conformers, with different populations, change in response to the different IL solutions.^39^ We consider that the variability in interaction energy between ions and proteins provides the possibility that some conformations may be strongly preferred while others are inhibited.^40^ Hence, we conducted temperature variable CD experiments, in order to probe the energy landscape of avidin and study the role of ion–protein interactions in determining the conformational preferences of avidin in ILs. These were combined with thermodynamic calculations to study the pathways and the conformational transitions and preferences of unmodified and modified avidin in low and high concentrations of ILs. It should be noted that while variable temperature CD spectroscopy is a well-established technique in structural biology for analysing proteins in different solutions,^9,17^ the technique is limited when studying ILs, as UV analysis showed that ILs absorb in the far UV (190–260 nm) region and distort the protein sample signal (see ESI Table S2†); limiting the ILs which we could examine using CD spectroscopy to [Cho][Hex] and [Cho]Cl.

**Fig. 3:**
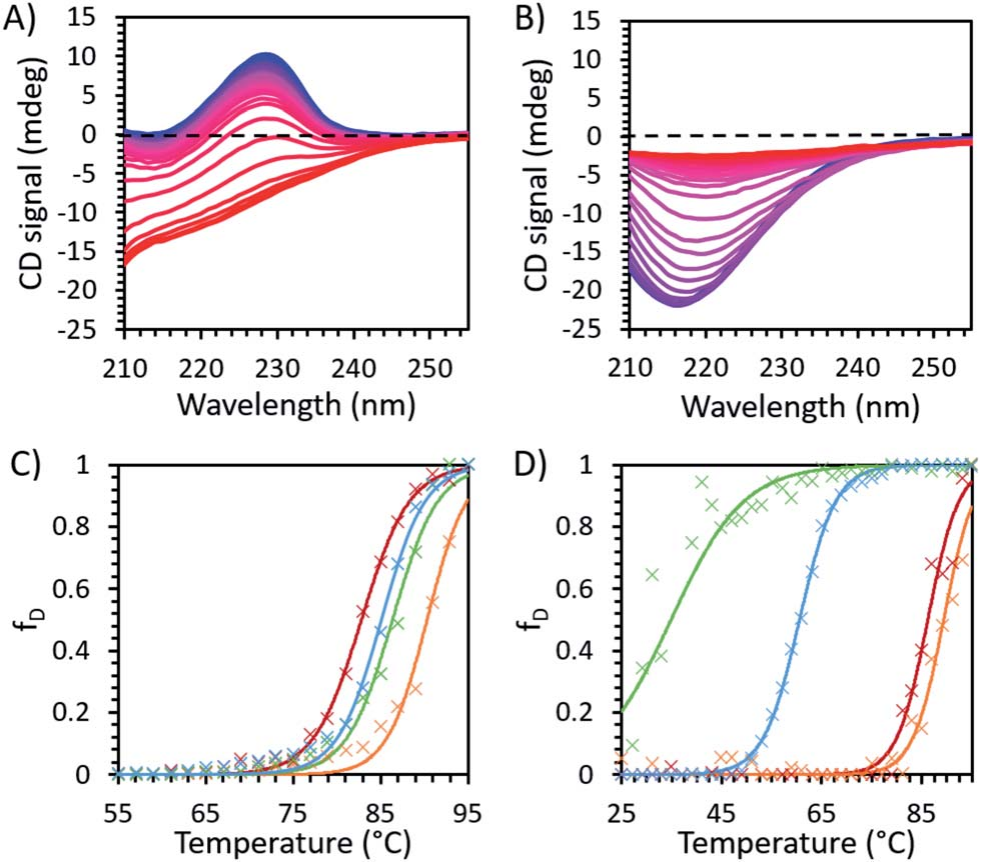
Temperature dependent CD spectroscopy data for (A) unmodified and (B) modified avidin in 30 wt% [Cho]Cl recorded at 2 °C intervals between 25 °C (blue) and 95 °C (red), showing the decrease in peak intensity with increasing temperature, corresponding to loss of secondary structure in avidin. The fraction of protein denatured in IL, with raw data (×) and fitted Boltzmann distribution curves (smooth lines) are shown in (C) unmodified avidin and (D) modified avidin in [Cho]Cl 50 wt% (green) and 30 wt% (blue), and 30 wt% [Cho][Hex] (red) and 10 wt% (orange) (see ESI Fig. S6 for CD fitting data and Method S1 for calculation methods†).

The CD spectra of unmodified and modified avidin in [Cho][Hex] and [Cho]Cl showed a loss of secondary structure with increasing temperature (Fig. 3). Specifically, for both unmodified and modified avidin, we observed temperature dependant conformational changes, with a high proportion of β-strand conformations at lower temperatures, shifting to an increasing α-helical population at high temperatures. In 10 wt% [Cho]Cl, unmodified avidin exhibited a positive peak at 228 nm and a negative peak at approximately 218 nm, corresponding to the native avidin β-sheet secondary structure.^15^ For modified avidin, the former is not visible, while the latter is significantly increased in breadth and intensity, likely due to an increase in α-helical structure, with additional negative peaks at 208 nm and 222 nm.^41^ We suggest that with increasing temperature, an increase in non-specific interactions between the IL solution molecules and avidin surfaces leads to exposed protein surfaces and disrupted secondary structure, which manifests as the loss of secondary structure in the range of 80 < *T*_m_ < 90 °C (Fig. 3).

**Fig. 4:**
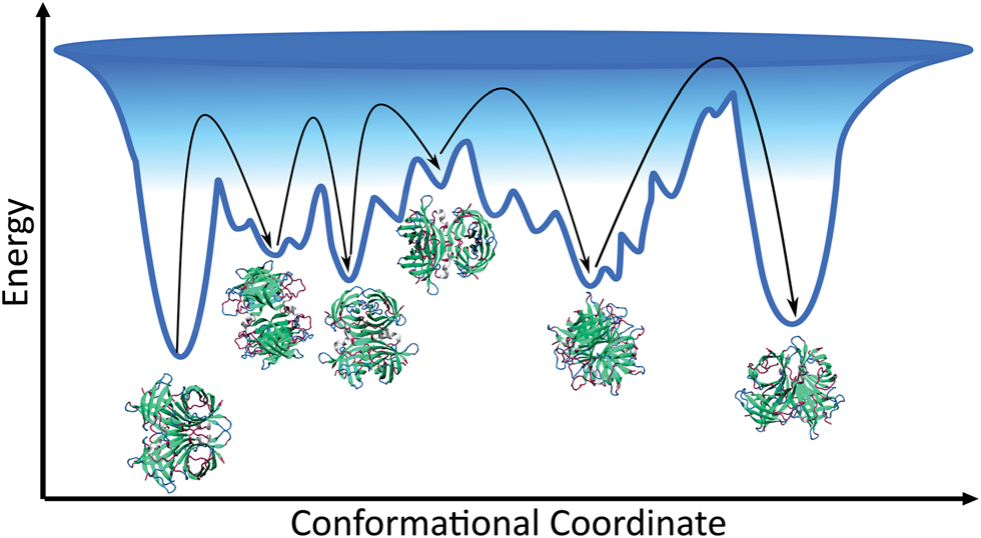
Schematic of the conformational space avidin can explore, showing the ensemble of avidin states and different minima of varying depth separated by kinetic barriers. Each minimum corresponds to a conformational state of avidin in IL solution, with ions excluded from schematic. The deeper the minima, the more stable the conformation of avidin, and the deepest minimum is associated with avidin in its native, initial state. The interactions between the IL and avidin lead to a rugged surface, with non-native local minima in which avidin conformers can get trapped. As represented by black arrows, avidin can explore conformational space until becoming trapped in a local minimum surrounded by high energy barriers it cannot overcome.

Given that the structure of avidin facilitates the entry of biotin into its binding pocket, the thermodynamically stable conformation of avidin is proposed to be a more flexible and more open conformation.^42^ In line with this, the energy landscape of unbound avidin in IL can be described as rugged, with avidin largely lacking specificity for a single compact conformation, resulting in a heterogenous ensemble of conformations of different stabilities and disordered regions (Fig. 4).^4^ Smoothing the landscape can be described by the drive of avidin to remove hydrophobic surfaces from contact with the IL, which would reduce the free surface energy.^5^

We observed higher thermostability of unmodified avidin in 10 wt% [Cho][Hex] (*T*_m_ 90.3 °C) compared to 30 and 50 wt% [Cho]Cl (*T*_m_ ∼ 85 °C), 30 wt% [Cho][Hex] (*T*_m_ 82.8 °C), and water (*T*_m_ ∼ 67 °C). Furthermore, while increasing the concentration of [Cho][Hex] reduced the thermostability of unmodified avidin, increasing the concentration of [Cho]Cl showed no impact on thermostability. In the framework of the energy landscape, we propose two phenomena are observed in this work, depending on the IL composition and concentration used. In the first case, the IL ions facilitate conformational transitions *via* energetically favourable interactions, or by stabilising partially folded states. The protein fluctuates between multiple potential energy minima, overcoming free energy barriers between them, toward a thermally stable conformation.^5^ In the second case, the IL ions promote conformations susceptible to denaturation, usually due to weak non-specific interactions, and the protein becomes rigid and kinetically trapped by a barrier and is separated from a more thermodynamically favourable and thermally stable conformation.^39,43^ In both cases, the extent to which the avidin conformers resemble the native state depends on the initial secondary structure of avidin and the experimental conditions, including temperature, ionic strength, pH, and solution concentration. Notably, in different IL solutions, the native contacts of avidin entered in competition with a vast number of alternative inter-molecular interactions that increased the roughness of the avidin energy landscape. A rougher surface, with a larger number of relatively shallow energy minima, as well as a rough surface within the deeper energy minima, would cause more hindrance for avidin to explore alternative conformational states, which are less kinetically accessible (Fig. 4).^44^ Thus, our findings emphasise that not only the depth and the presence of energy minima, but also the roughness of the energy surface dictates the stability of avidin.

By performing thermodynamic calculations, we can begin to correlate the conformational preferences to the structure of avidin, and gain insight into how the ions associate with avidin in relation to the bulk solution. It should be noted that due to *T*_m_ approaching the maximum temperature that could be measured without the solvent boiling (95 °C), the calculation uncertainties in Δ*H*_m_ and Δ*S*_m_ were relatively large. Nonetheless, while the denatured state was approximated from a small number of data points, the trend was consistent between the samples and the margin of error was consistent.

It is expected that with increasing IL concentration, the increase in conformational diversity and disorder of the native, initial state would be such that the entropy gain associated with denaturation would decrease.^45^ Accordingly, our calculations for unmodified avidin showed that Δ*H*_m_ and Δ*S*_m_ decreased with increasing IL concentration (Table 3), exemplified by the case of 30 wt% [Cho][Hex] and 50% [Cho]Cl. This may also be due to molecular confinement effects discussed earlier, involving the local and long-range contribution of the ion–protein interactions, as the motions of the most flexible domains of avidin and the IL molecules are restricted. We found that the values of Δ*H*_m_ for modified avidin were 40–70% of the values observed for unmodified avidin, indicating that overall the thermal barrier to denaturation for modified avidin was lower. In this context, compared to unmodified avidin, the intrinsically more highly disordered modified avidin has an energy surface that contains many local energy minima separated by relatively low energy barriers, ensuring transition between different conformational states. Specifically, for modified avidin, the values of Δ*H*_m_ and Δ*S*_m_ increase with increasing IL concentrations, with more pronounced changes observed in [Cho]Cl than [Cho][Hex], supporting that higher concentration is linked to greater entropy and larger free energy, where avidin can adopt a wide range of different conformations with different orientations and arrangements of its helices. It can be suggested that the significant increase in entropy change for avidin denaturation with increasing IL concentration in [Cho]Cl (1800 J K^−1^ mol^−1^ for 50 wt% [Cho]Cl compared to 760 J K^−1^ mol^−1^ for 30 wt%), came from the greater flexibility of the helical loop of modified avidin in 50 wt% [Cho]Cl. Furthermore, reduction in *T*_m_ of over 50 °C was observed in this case, compared to the modified protein in either concentration of [Cho][Hex], implying avidin could explore conformational space, sampling a wide conformational area and overcoming energy barriers, until becoming trapped in a free-energy minimum, away from the initial native conformation. This observation highlights that not all avidin systems are created equal, with some ensembles being more heterogeneous than others, and different ensembles exhibiting preferences for particular conformations with different thermostabilities.

**Table:** The thermodynamic data calculated from the CD experiments for unmodified and modified avidin in [Cho]Cl and [Cho][Hex]

IL	Unmodified			Modified		
	*T*_m_ (°C)	Δ*H*_m_ (kJ mol^−1^)	Δ*S*_m_ (J K^−1^ mol^−1^)	*T*_m_ (°C)	Δ*H*_m_ (kJ mol^−1^)	Δ*S*_m_ (J K^−1^ mol^−1^)
[Cho]Cl 30%	85.1 ± 0.1	101 ± 7.2	1180 ± 49	60.6 ± 0.1	46 ± 4.3	760 ± 42
[Cho]Cl 50%	86.4 ± 0.2	88 ± 22	1020 ± 150	34.9 ± 0.9	54 ± 6.6	1800 ± 128
[Cho][Hex] 10%	90.3 ± 0.2	114 ± 29	1260 ± 187	89.4 ± 0.3	58.9 ± 7.0	656 ± 45
[Cho][Hex] 30%	82.8 ± 0.1	92.4 ± 2.1	1110 ± 26	86.2 ± 0.3	61.3 ± 19	709 ± 127

**Fig. 5:**
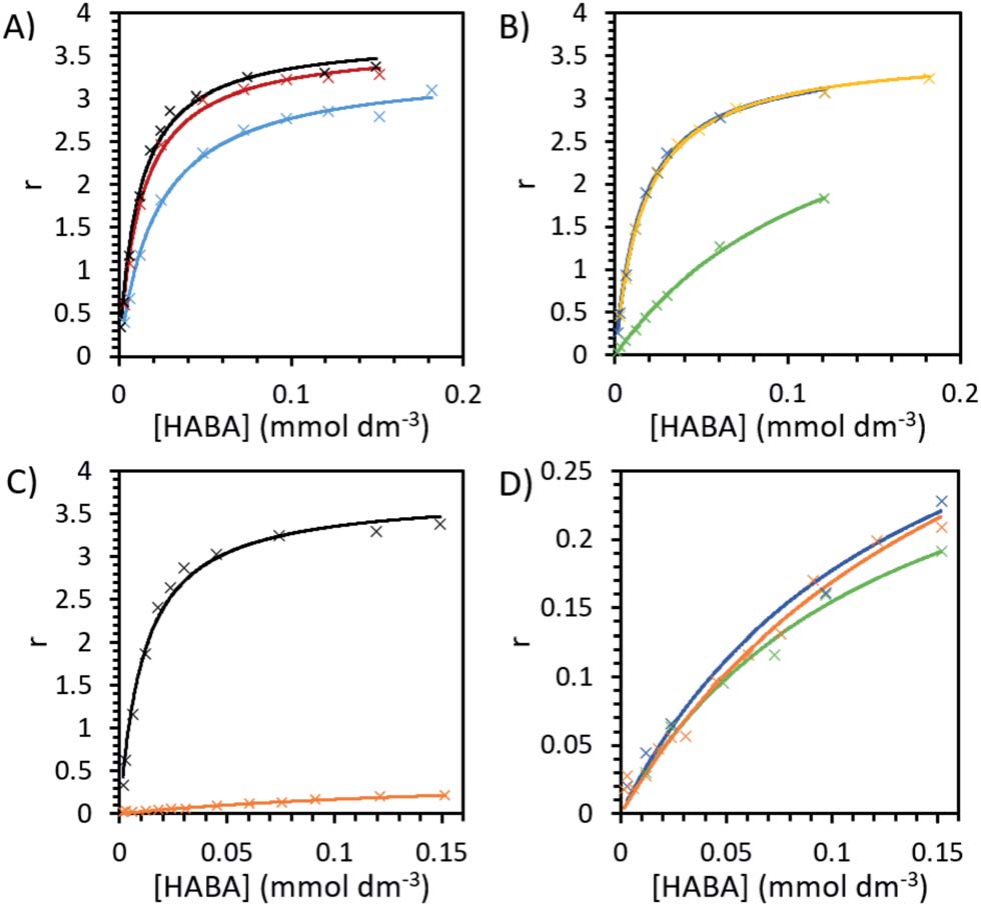
The binding plots of HABA concentration against saturation function, *r*, ratio of bound HABA to avidin molecules, with a maximum value of 4. HABA with unmodified avidin in (A) 0.1 M pH 6.8 phosphate buffer (black), 0.1 M pH 5.1 citrate–phosphate buffer (red) and 0.1 M pH 9.1 tris buffer (blue) and (B) 10 wt% [Cho][Asp] (yellow), [Cho][DHP] (dark blue) and [Cho]Cl (green). The binding plots for HABA (C) in pH 6.8 phosphate buffer with unmodified avidin (black) and modified avidin (orange) and (D) with modified avidin in phosphate buffer (orange), 10 wt% [Cho][DHP] (dark blue) and 10 wt% [Cho]Cl (green).

Binding experiments were conducted for unmodified and modified avidin in various solvents with azo-dye HABA, a known, weak ligand for avidin, in order to study the effects of different ILs and possible changes in binding affinity *via* conformational shifts.^46^ Solutions containing 10 wt% of each of our choline-ILs were tested, as well as 0.1 M solutions of pH 5.1 citrate–phosphate buffer, pH 6.8 phosphate buffer and pH 9.1 and 11 tris buffer. HABA–avidin binding was monitored by UV-Visible (UV-Vis) spectroscopy, with the concentration of the HABA–avidin complex determined from a peak at 500 nm (*ε*_500_ = 34 500 M^−1^ cm^−1^; see ESI Fig. S7† for example of raw UV-Vis data). The approximate values of the binding dissociation constant, *K*_d_, and the number of available binding sites, *n*, were determined by applying non-linear regression and the Hill–Langmuir equation to plots of the saturation constant (the ratio of concentration of bound HABA to avidin molecules) against the known concentration of HABA added (Table 4 and Fig. 5).^47^

**Table:** The extrapolated *K*_d_ and *n* values from the binding plots of unmodified avidin in the 6 solutions tested which exhibited binding; three buffer solutions and three aqueous choline salts

Solution	Unmodified avidin		Modified avidin	
	*K*_d_ (×10^−5^ mol dm^−3^)	*n*	*K*_d_ (×10^−5^ mol dm^−3^)	*n*
pH 6.8 phosphate buffer	1.1 ± 0.1	3.7 ± 0.1	18 ± 5	0.5 ± 0.1
pH 5.1 citrate–phosphate buffer	1.3 ± 0.1	3.65 ± 0.06	—	—
pH 9.1 tris buffer	2.2 ± 0.2	3.39 ± 0.06	—	—
10 wt% [Cho][Asp]	1.7 ± 0.1	3.56 ± 0.05	—	—
10 wt% [Cho]Cl	14 ± 1	3.9 ± 0.2	13 ± 3	0.35 ± 0.5
10 wt% [Cho][DHP]	1.6 ± 0.1	3.53 ± 0.05	14 ± 6	0.4 ± 0.1

In order to systematically examine whether avidin undergoes substantial, environmentally dependent conformational transitions, converting among conformers under different pH conditions, we first analysed buffer environments as controls. In buffer solution with pH 6.8 (0.1 M phosphate), at very low HABA concentrations the amount of bound HABA increased rapidly, plateauing with maximal value in this range of approximately 84% of binding sites occupied (Fig. 5). In reasonable agreement with the literature, the observed *K*_d_ of avidin was 1.13 × 10^−5^ mol dm^−3^ and *n* was a reasonable approximation to 4, the number of binding sites on each avidin molecule.^19,48^ Binding in buffer solution with pH 5.1 (0.1 M citrate–phosphate), was nearly as efficient as in pH 6.8, indicating good structural preservation of the binding sites. At pH 9.1 (0.1 M tris buffer) this decreased by approximately 10%, with maximal binding site occupancy in this range at 71%, and in pH 11 (sodium hydroxide solution) no binding at any HABA : avidin ratio was observed. It should be noted that this is caused at least in part by ligand effects. HABA has two tautomeric structures, an azo-form and a hydrazone form, only the latter of which is known to bind to avidin.^49^ The p*K*_a_ of the phenolic proton in HABA is approximately 8.2. At pHs above this, HABA will exist primarily as a dianion in the azo-form and avidin–HABA binding is reduced, attributed to the additional energy required to convert the dianion to the hydrazone form for binding.^50,51^ This is consistent with our observation of decreased, but still substantial binding in pH 9.1 control buffer solution. It should be noted that the p*K*_a_ of the hydrazone proton is also anticipated to be around 10, so at high pHs the binding hydrazone tautomer is unable to form and binding cannot occur.^52^

For unmodified avidin, we observed no binding in 10 wt% [Cho][Met] and [Cho][Phe], for which the solution pHs were known to be >10.5. This would be influenced by the pH effect described above, and hence it was not possible to discern the effect of the ions in these ILs on protein–ligand interactions. A total lack of binding was also observed in 10 wt% [Cho][Ger] and [Cho][Hex], respectively, the pH of both of which were 8–8.5 (see Table 2) which could decrease binding but would not be inhibitory. However, it is well established in the literature that ligand binding in avidin is dependent on hydrophobic interactions of residues in the binding site.^49^ In both [Cho][Hex] and [Cho][Ger], the anion hydrocarbon chain would be reasonably hydrophobic. It is therefore possible that interactions between the anion and the avidin binding site affected the conformation of avidin such that it could no longer bind HABA. This is further supported by the findings of the CD experiments, where in [Cho][Hex] avidin conformational change was evidenced by the increase in *T*_m_ (Fig. 3).

While it is unlikely that major structural changes occurred in the avidin binding site, it is probable that as ions interacted with specific residues, local disturbances indirectly affected the binding site.^29^ For example, the ions could disrupt the structuring water molecules surrounding the protein surface and raise the entropy of flexibility of the protein, leading to lower binding between avidin and ligand.^53^ In [Cho][DHP] and [Cho][Asp], the binding curves were of similar amplitude and curvature to buffer, with maximal binding site occupancy of 78% and 81%, respectively, achieved in the range tested; yet, in [Cho]Cl the binding curve slope was significantly shallower, with maximal occupancy just 53% and *K*_d_ increased by more than a factor of 10 to 14 × 10^−5^ mol dm^−3^, indicating weaker binding (Table 4 and Fig. 5). It is worth considering that despite significantly weaker binding observed, unmodified avidin in [Cho]Cl showed higher thermostability, independent of [Cho]Cl concentration. Thus, the specific interactions between the ions and protein residues, whether transient or permanent, affected the electrostatic energies of the protein and could steer it to alternative conformations.^5,29^ On the basis of this, we propose that the available conformational space for avidin in 10 wt% [Cho]Cl becomes less restricted as molecular confinement is reduced, and lower binding corresponds with more conformationally heterogenous ensembles.

Modified avidin in pH 6.8 phosphate buffer showed weaker binding (*K*_d_ = 18 ± 5 × 10^−5^ mol dm^−3^) and *n* = 0.5 ± 0.1, indicating that a large proportion of binding sites were no longer active following modification (Fig. 5). Values of *n* in IL solutions were slightly reduced but comparable to buffer, indicating the ILs were not able to significantly alter the number of active binding sites. Likely, the presence of excess surfactant (verified by SAXS data), precluded the ligand from binding to avidin.^33,54^ To overcome this, one mechanism proposed is for the IL to effectively compete with existing hydrogen-binding interactions and separate the avidin and surfactant.^28,34^ However, due to the strong interactions involved, this was not possible for the ILs examined. We focus our analysis on modified avidin in 10 wt% [Cho][DHP] and [Cho]Cl, for a direct comparison with the data obtained for unmodified avidin. Binding in both [Cho]Cl (*K*_d_ = 13 ± 3 × 10^−5^ mol dm^−3^, *n* = 0.35 ± 0.5) and [Cho][DHP] (*K*_d_ = 14 ± 6 × 10^−5^ mol dm^−3^, *n* = 0.4 ± 0.1) was slightly stronger than but comparable to the pH 6.8 buffer. Given that ILs decreased binding strength of unmodified avidin, but slightly increased it for modified avidin, it can be suggested that the ions are likely essential mitigators, coordinating to the charged surface residues of avidin to counteract adverse modification effects and enhance binding strength.^55^

It is worth noting that the *K*_d_ in 10 wt% [Cho]Cl was the same for the unmodified and modified avidin (within the range of uncertainty), while in all other solutions the *K*_d_ of modified avidin was significantly lower. Additional evidence for a similar IL-mitigating temperature dependence is also demonstrated by the decrease in thermostability observed for modified avidin in 30 wt% and 50 wt% [Cho]Cl compared to in other solutions examined. The reduced thermostability is attributed to a shift in the conformational landscape of modified avidin. It can be suggested that when the binding site was not flexible enough, there was a trade-off between avidin thermostability and conformational stability, in order to preserve the structure of the binding site and maintain high ligand–protein affinity; however this hypothesis requires further investigation.^3,46^

Given that avidin can bind to HABA and biotin, it can likely explore a larger conformational space compared to a protein that can only bind to a single ligand.^4,56^ In order to demonstrate that ILs can cause a population shift in the conformational landscape of avidin, the biotin ligand, a stronger ligand for avidin than HABA (*K*_d_ = 10^−15^ compared to 6 × 10^−6^, respectively), was used to study possible competitive inhibition for substrate binding.^48^ By performing a titration of the bound complex with biotin, the amount of HABA displaced was monitored by UV-Vis spectroscopy, and the inhibition constant, *K*_i_, for biotin with the avidin–HABA complex was calculated (Fig. 5 and Table 5).^46,48^ As expected, the effect of IL on binding with a strong ligand was less pronounced than with a weak ligand (for example, a factor of 5 difference between buffer and [Cho]Cl in biotin binding compared to factor of 13 for HABA).

**Table:** The calculated values of *K*_i_ for biotin with HABA–avidin, accounting for different HABA binding strengths

Conditions	*K*_i_ (mol dm^−3^)
pH 6.8 phosphate buffer	5.0 × 10^−7^
[Cho][Asp]	5.4 × 10^−7^
[Cho]Cl	2.5 × 10^−6^
[Cho][DHP]	7.6 × 10^−7^

**Fig. 6:**
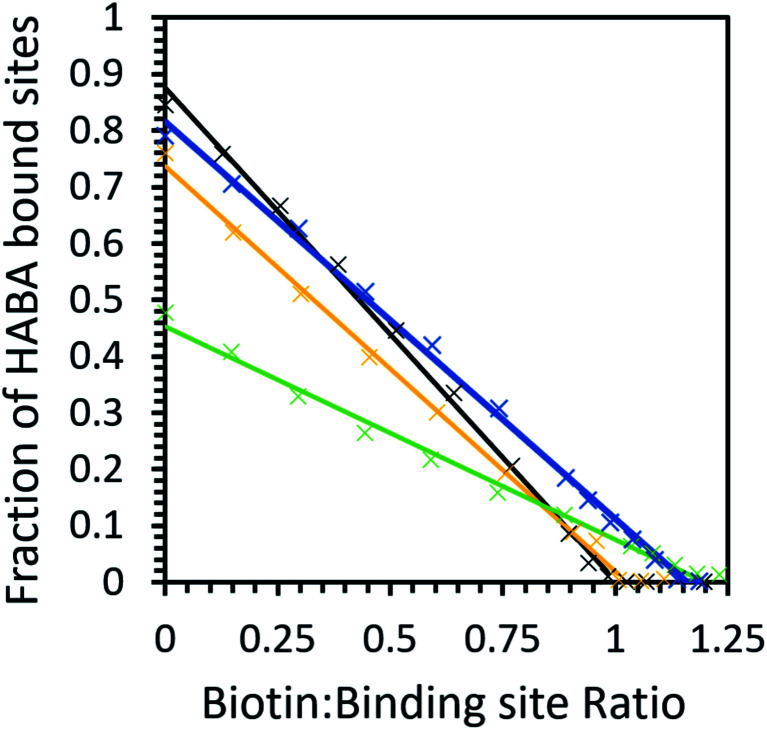
The biotin displacement of HABA in phosphate buffer (black), 10 wt% [Cho][Asp] (yellow), 10 wt% [Cho][DHP] (dark blue) and 10 wt% [Cho]Cl (green) with data (×) and a linear fit (solid line). Samples have an initial HABA : binding site ratio greater than 10 : 1.

The competitive binding study showed that in buffer and [Cho][Asp], HABA was fully displaced at a 1 : 1 biotin : binding site ratio, indicating all biotin molecules occupied the avidin binding sites. However, in [Cho]Cl and [Cho][DHP], a small excess of biotin was needed (1.2 : 1 ratio) before all of the HABA was displaced (Fig. 6), demonstrating that biotin binding of unmodified avidin was weaker in these ILs.^48,57^ It is also worth noting that, despite HABA not occupying all binding sites at the start of the displacement study, HABA was displaced from binding sites, at a constant rate, in each IL. This shows biotin bound to both the empty and HABA-occupied sites simultaneously and proportionally under all conditions tested. Therefore, in all cases where dynamic conformations were induced by ILs, the ability of avidin to bind biotin at both empty and occupied sites, with little distinction between the two, was retained, regardless of any excess ligand required.

Our calculations show that the biotin–avidin *K*_i_ was significantly higher in [Cho]Cl compared to buffer, indicating weaker avidin–biotin binding which was unsurprising given the weaker HABA binding observed in this IL. Most notably, [Cho][DHP] exhibited slightly stronger HABA binding than [Cho][Asp], but inhibition by biotin was less effective in the former case than the latter. These results demonstrate that the ILs did not directly inhibit the binding site; and we propose that the interactions between the ions and protein facilitated avidin to span a large conformational space and participate in diverse interaction scenarios, for example, allowing sampling of rare conformers and enhanced affinity for HABA relative to biotin.^32,58^ The relative affinity for HABA in [Cho][DHP] increasing while the relative affinity for biotin decreased is possibly the strongest piece of evidence from the binding studies for avidin experiencing IL-induced conformational changes, providing quantifiable data on the ‘invisible’ avidin conformer, rarely populated in solution. Therefore, by introducing favourable ion–protein interactions, reducing the loss of conformational entropy, and optimising direct interactions between avidin and HABA, unmodified avidin could adopt a less probable conformation of the whole ensemble with higher affinity for HABA, similar to modified avidin in [Cho][DHP].

## Conclusions

In this work we examined the thermal stability and conformational preferences of unmodified and modified avidin in different choline-based ionic liquids and buffer solutions. We studied the effects of ILs on the conformational transitions of avidin using DLS, SAXS, DSF, CD spectroscopy, and binding experiments. We showed that ILs can conformationally stabilise avidin, as was the case for unmodified avidin in up to 50 wt% [Cho]Cl and dilute [Cho][Hex] and [Cho][Asp]. However, ILs can also drive avidin to a new local free energy minimum on the energy landscape, and molecular confinement by ILs could lead to reduced thermal protein stability and binding, as exemplified by unmodified avidin in 30 wt% [Cho][Hex] and modified avidin in [Cho]Cl. Since a wide range of combinations of anions and cations of ILs are available, it is not surprising that different ILs exerted distinct effects on the structure and thermal stability of avidin, driven by the nature of the ions and the interactions with the protein, and in some cases limited the conformational space sampled by avidin. While it was not possible to solve the specific structures of the avidin conformers, possibly the avidin surface residues and the loop structure^59^ showed different degrees of flexibility in response to ILs and ligands. The experimental results also uphold an important prediction of energy landscape theory, that different folding pathways may become dominant under different folding conditions. Therefore, the tunability of ILs could be of great value to understanding the conformational energy landscape of more complex systems, and where environmental factors result in the interplay of different conformations, we can use ILs to uncover and stabilise specific conformational states from a heterogeneous conformational ensemble.

## Experimental section

### Materials

Lyophilised avidin from hen egg white was purchased from Merck and stored at −18 °C. Choline hydrogen carbonate ([Cho][HCO_3_]; 76.6% in water determined by titration), [Cho]Cl (>98%), [Cho][DHP] (>98%), hexanoic acid (99.8%), geranic acid (80% technical grade), tris(hydroxymethyl)aminomethane (tris) base, disodium phosphate (Na_2_HPO_4_), citric acid monohydrate, SYPRO orange dye (5000X concentrate in DMSO), *N*,*N*′-dimethyl-1,3-propanediamine (DMPA, >97%), glycolic acid ethoxylate lauryl ether *M*_n_ 690, 1-ethyl-3-(3-dimethylaminopropyl)carbodiimide (EDC, >97%) lyophilised biotin powder were purchased from Sigma (Sigma Aldrich Corp, St Louis, MO, USA) and stored as recommended. Geranic acid was recrystallised five times at −70 °C from a solution of 70 wt% geranic acid/30 wt% acetone, a known literature procedure.^60^ Methionine, phenylalanine, aspartic acid and HABA (all >98%) were purchased from Tokyo Chemical Industry (Tokyo Chemical Industry Co. Ltd, Tokyo, Japan) and used as received. Visking dialysis tubing with MWCO 12–14 kDa was purchased from Medicell (Medicell Membranes Ltd, London, UK). All water used was ultrapure water, obtained from a PURELAB Ultra water purifier (ELGA LabWater, High Wycombe, UK) with resistivity of 18.2 MΩ.

### IL synthesis and characterisation

[Cho][Hex], [Cho][Ger] and [Cho][Asp] were prepared from hexanoic, geranic and aspartic acids respectively as detailed in the literature.^61^ Briefly, each acid was slowly added to aqueous [Cho][HCO_3_], in a 1 : 1 ratio, with stirring at ambient temperature and pressure. Reaction vessels were left to stir overnight, then water was removed by rotary evaporation at 35 °C, and ILs were finally fried under reduced pressure (2 mbar) and 40 °C for 48 hours.

[Cho][Met] and [Cho][Phe] were synthesised by anion exchange with Amberlite IRN78 hydroxide form anion exchange resin. A dilute [Cho]Cl solution (30 mg mL^−1^) was prepared and added dropwise to a gravity column packed with 150 mL resin wetted-bed (approximately 5 equivalents) at a flow rate of 5 mL min^−1^, forming [Cho]OH *in situ* which was added dropwise to a round bottom flask containing the amino acid (phenylalanine or methionine) and water, which was cooled in an ice-bath with stirring. The column was washed through with 300 mL of ultrapure water and the collection vessel was stirred at 3 °C and ambient pressure for 24 hours in the dark. Water was removed by rotary evaporation at 30 °C and a 9 : 1 acetonitrile/methanol mixture was added to precipitate any unreacted amino acid. The mixture was filtered and the solvents were removed by further rotary evaporation at 30 °C. The resulting ILs were dried under reduced pressure (2 mbar) and 35 °C for 48 hours.

Aqueous solutions of [Cho]Cl and [Cho][DHP] were prepared by addition of ultrapure water to the salt, then shaking at room temperature for 1 hour to give 80 wt% IL solutions.

All ILs were characterised by ^1^H and ^13^C NMR spectroscopy in either DMSO-d^6^ or D_2_O at 25 °C using Bruker Advance III, 400 MHz spectrometer (Bruker Corporation, Billerica, MA, USA). Electrospray ionisation mass spectrometry was performed using Waters LCT Premier Mass Spectrometer (Waters Corporation, Milford, MA, USA) in positive and negative ion mode.

### Protein modification

Surface modification was performed as reported previously.^62^ Briefly, cationisation was performed by coupling acidic residues on avidin (2 mg) to DMPA (0.9 g, excess) in dilute aqueous solution at pH 5.8 using EDC (0.4 g). The filtered protein solution was dialysed against ultrapure water for 24 hours to remove excess reactants and byproducts and protein concentration was determined by UV-Vis spectroscopy on a Shimadzu UV-2600 (Shimadzu Corporation, Kyoto, Japan). The cationised protein solution was added dropwise to a *M*_n_ 690 glycolic acid ethoxylate lauryl ether solution, with a 5 : 1 surfactant: positive surface residue ratio (580 equivalents per avidin molecule). After further stirring (2 hours) and dialysis (48 hours), the solution was centrifuged at 4000 rpm for 30 minutes, the supernatant decanted and lyophilised and the residue thermally annealed at 60 °C until an off-white, viscous liquid formed.

DSC was performed on a TA Q2000 (TA Instruments, New Castle, DE, USA) to demonstrate the conjugation of the protein and surfactant. Samples were heated at 10 °C min^−1^ between −80 °C and 100 °C and cycled 4 times to erase the thermal history and achieve reliability. Decomposition temperatures were found by TGA on a NETZSCH STA 449 F5 Jupiter (NETZSCH Group, Selb, Germany). Samples in alumina pans were heated from 25 to 500 °C at 10 °C min^−1^ under 40 mL min^−1^ of N_2_ and decomposition onset temperature (*T*_d_) determined using Proteus Analysis software. DLS measurements were collected using a Malvern Zetasizer μV (Malvern Panalytical Ltd, Malvern, UK). Aqueous solutions with a protein concentration of 0.1–1.0 mg mL^−1^ were measured in disposable cuvettes at 25 °C distributions were formed from the accumulation of ten 30 second experiments.

### Small angle X-ray scattering

Samples of appropriate IL and concentration were prepared from pure IL and ultrapure water and transported under cold conditions (2–8 °C) to Diamond Light Source (Oxford, UK). SAXS experiments were carried out on either the B21 or I22 beamlines. Protein samples were 5 mg mL^−1^ for unmodified avidin and 3.5 mg mL^−1^ for modified avidin on B21 beamline and 8 mg mL^−1^ for all protein on I22 beamline. Data was averaged, background subtracted and analysed using ScÅtter software by SIBYLS beamline, Advanced Light Source (Berkeley, CA, USA). SasView software (http://www.sasview.org/) was used to more accurately determine the *R*_g_ of the protein in each sample by fitting the data using set parameters and models. The SLD values of the protein, modified protein, surfactant and solvent were calculated from the molecular formulae and density using the online SLD calculator by NIST (Gaithersburg, MD, USA) (https://sld-calculator.appspot.com/). Plots were fitted over at least 400 data points, with fits optimised by minimising Chi squared for realistic parameter values.

### Differential scanning fluorimetry

Tris buffer (0.15 M, pH 9.1) was prepared using tris base and adjusted with HCl. Citrate–phosphate buffers (0.15 M, pH 5.1 and 7.2) were prepared using a 2 : 1 ratio of disodium phosphate and citric acid monohydrate and adjusted with NaOH.

Protein-IL and protein-buffer samples (45 μL) were prepared with 2.0 mg mL^−1^ avidin and pipetted into a 96-well plate. SYPRO orange aliquots were diluted 25 times with ultrapure water and 5 μL added to each sample, for a final SYPRO orange concentration of 20X, and the plate was sealed with adhesive film. Samples were run on an Eppendorf Mastercycler ep realplex quantitative cycler (Eppendorf, Hamburg, Germany) with a 25–99 °C temperature ramp, 0.5 °C resolution and 4 °C min^−1^ heating rate, followed by a 30 s hold at 99 °C. Fluorescence was measured at 570 nm, the emission maximum of SYPRO orange. Spectra for protein-free background samples of each IL were subtracted from the sample spectra. The data was smoothed using Origin Savitzky–Golay method with 7 points and polynomial order 5 (OriginLab Corporation, Northhampton, MA, USA). *T*_m_, Δ*S*_m_ and Δ*H*_m_ were calculated from the raw fluorescence data (see ESI Fig. S5 and S6†) by assuming a two-state denaturation process for avidin (see ESI Method S1† for detailed method).

### Circular dichroism

Temperature controlled CD spectra were recorded at 2 °C intervals between 25–95 °C on an Applied Photophysics Chirascan Spectropolarimeter (Applied Photophysics Limited, Leatherhead, UK) at Diamond Light Source on beamline B23 with 1 point per 1 nm, 1 s per point from 190–270 nm for [Cho]Cl and 210–260 nm for [Cho][Hex]. Background spectra for the IL solutions were recorded at room temperature and subtracted from the temperature variable data. Background normalisation was achieved by setting the average ellipticity of the 263–270 nm region to zero. Data was smoothed and *T*_m_, Δ*S*_m_ and Δ*H*_m_ were calculated as for DSF.

### HABA binding

Excess HABA was added to buffer/water, the solution shaken for 2 hours then filtered using a 0.22 μm polyethersulfone filter to remove undissolved HABA. HABA concentration was determined by UV-Vis spectroscopy with extinction co-efficient *ε*_350_ = 20 500 M^−1^ cm^−1^.^63^ IL was added to aqueous HABA solutions to give 10 wt% IL solutions of known HABA concentration. Modified and unmodified avidin (800 μL, 0.2 mg mL^−1^) samples were prepared in buffer or 10 wt% IL and known amounts of HABA in the corresponding solution were added, UV-Vis spectra recorded, then additions repeated until approaching saturation. Degree of binding was calculated from the absorbance at 500 nm and *ε*_500_ = 34 500 M^−1^ cm^−1^ for bound HABA. Reference samples (equal amount HABA solution in the buffer or IL) were recorded simultaneously and the spectra subtracted. Values of dissociation constant *K*_d_ and number of binding sites *n* were calculated from plots of HABA concentration *vs.* saturation function *r*, with 
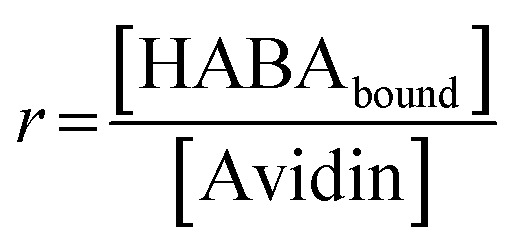
 and fitting using the enzyme kinetics non-linear regression on origin.

### Competitive biotin/HABA binding

Bound HABA–avidin samples with initial HABA : binding site ratio at least 10 : 1 were titrated with a 0.5 mM biotin solution and the decrease in absorbance at 500 nm measured by UV-Vis Spectroscopy. Biotin concentration was plotted against *r* to calculate the IC_50_ of biotin, the half maximal inhibitory concentration and hence the *K*_i_ (inhibition constant) for avidin–biotin binding in the presence of HABA.

## Conflicts of interest

There are no conflicts to declare.

## Supplementary Material

SC-012-D0SC04991C-s001
