# Supplementary material for: Exploring conformational preferences of proteins: ionic liquid effects on the energy landscape of avidin
Source: Chem Sci. 2020 Oct 23;12(1):196–209. doi: 10.1039/d0sc04991c (PMC8178808; doi:10.1039/d0sc04991c)
Supplement: SC-012-D0SC04991C-s001 [file SC-012-D0SC04991C-s001.pdf]

Electronic Supplementary Material (ESI) for Chemical Science.
This journal is © The Royal Society of Chemistry 2020

Exploring conformational preferences of proteins: Ionic liquid effects on the energy landscape of avidin

Talia A. Shmool,§[a] Laura K. Martin,§[a, b] Coby J. Clarke,[a] Liem Bui-Le,[a] Karen M. Polizzi,[a, b] and Jason P. Hallett*[a]

* Corresponding author: **Email**: j.hallett@imperial.ac.uk. **Phone**: +44 (0)20 7594 5388

[a] Department of Chemical Engineering, Imperial College London, London SW7 2AZ, United Kingdom

[b] Imperial College Centre for Synthetic Biology, Imperial College London, London SW7 2AZ, United Kingdom

§ These authors contributed equally to this work

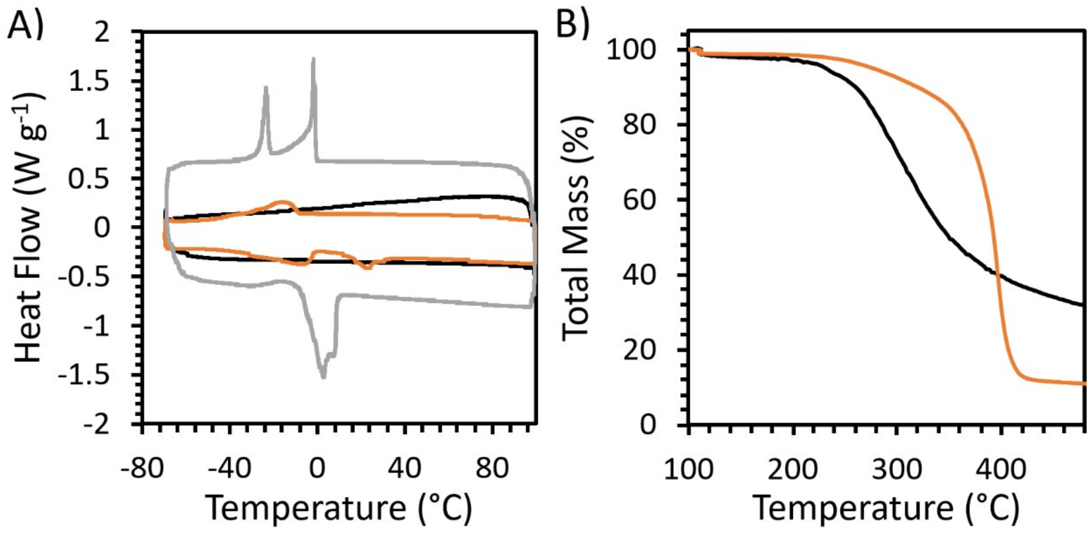

Figure S1: A) DSC spectra recorded between -80 °C and 100 °C at 10 °C min^{-1} for unmodified (black) and modified (orange) avidin and the M_n 690 glycolic acid ethoxylate lauryl ether surfactant (grey). Four full cycles were recorded to erase the thermal history, and cycles three and four are shown to emphasise reliability of the data. Two transitions are observed in the heating (bottom) curve for the modified avidin, as in the literature (Perriman, A.W.; Cölfen, H.; Hughes, R. W.; Barrie, C. L.; Mann, S. Solvent-Free Protein Liquids and Liquid Crystals. *Angew. Chem. Int. Ed.* 2009, 48 (34), 6242-6246. https://doi.org/10.1002/anie.200903100). The first transition (-11.7 °C) is in reasonable agreement with the melting transition observed for the surfactant, while the second is unique to the modified protein. We therefore attribute the second transition at 23.7 °C to the melting of modified avidin. For unmodified avidin no transitions are seen, as would be expected since the temperature range remains below decomposition (as shown in B) and the DSC was not be sensitive enough to detect denaturation. B) TGA spectra of unmodified (black) and modified (orange) avidin at 10 °C min^{-1}. The full spectrum was recorded between 25-500 °C, with samples held at 100 °C to remove water before decomposition.

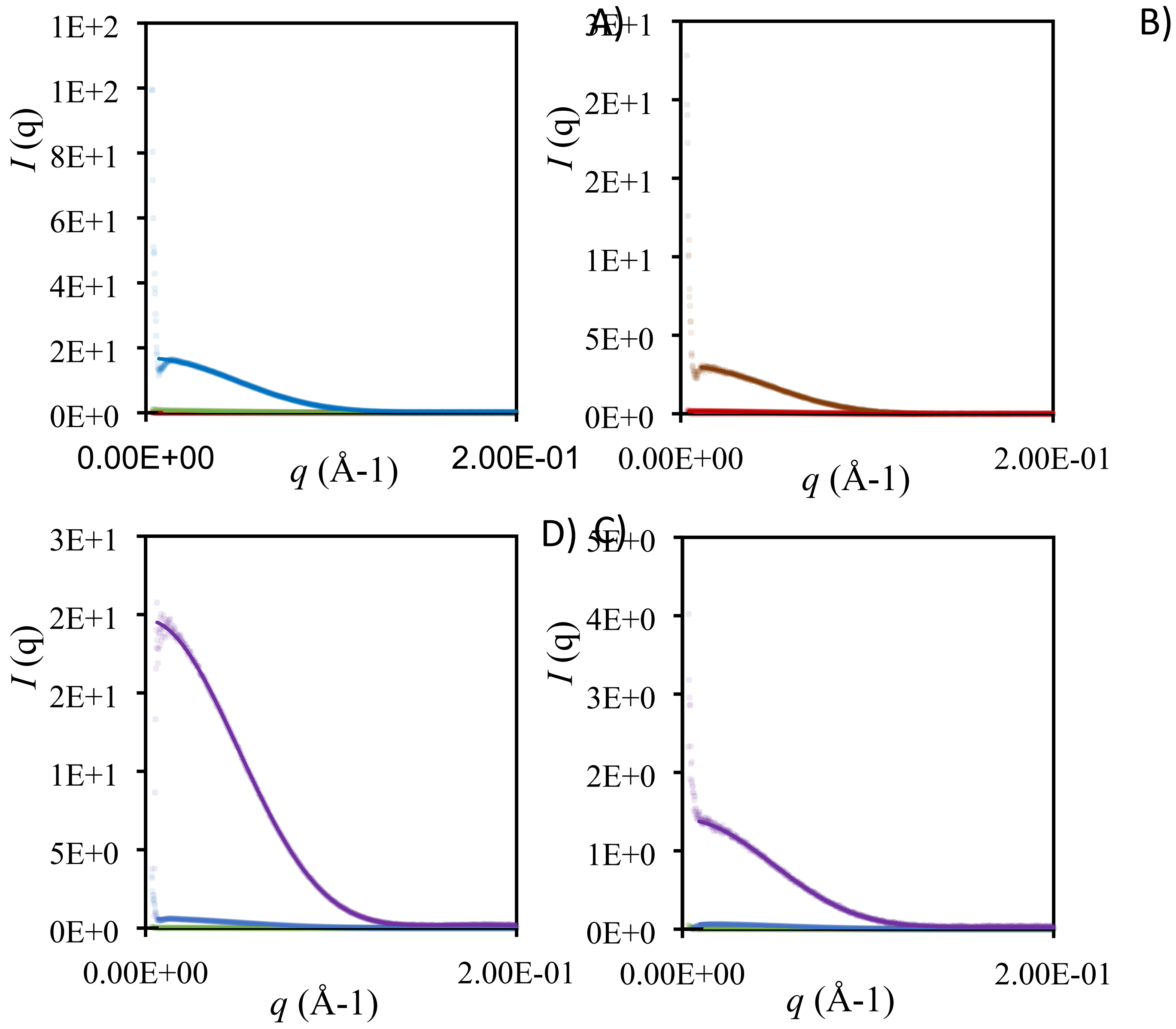

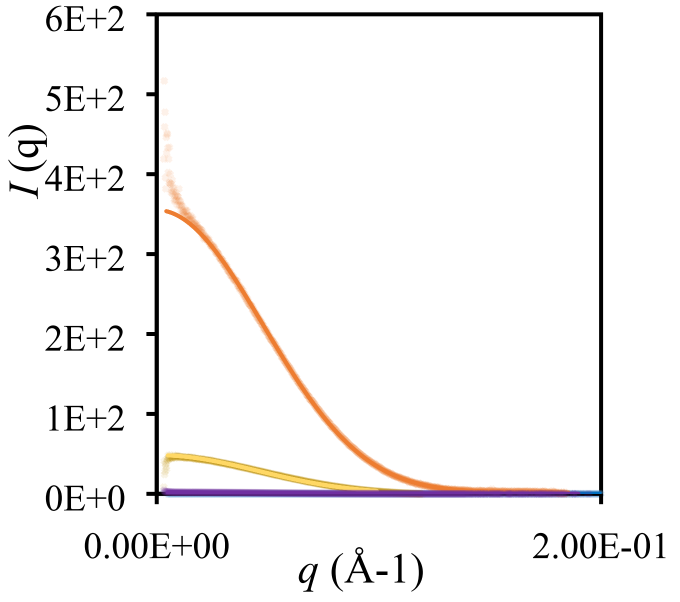

Figure S2: SAXS data (x) and fit (solid line) of unmodified avidin in all aqueous solutions: A) pH 5.1 citrate-phosphate buffer (red), pH 7.2 phosphate buffer (green), pH 9.1 tris buffer (blue). B) 10 wt% [Cho]Cl (pink), 50 wt% [Cho]Cl (red), 10 wt% [Cho][DHP] (brown). C) 10 wt% [Cho][Asp] (green), 10 wt% [Cho][Met] (blue), 10 wt% [Cho][Phe] (purple). D) 50 wt% [Cho][Asp] (green), 50 wt% [Cho][Met] (blue), 50 wt% [Cho][Phe] (purple). E) 10 wt% [Cho][Hex] (blue), 50 wt% [Cho][Hex] (purple), 10 wt% [Cho][Ger] (yellow), 30% [Cho][Ger] (orange).

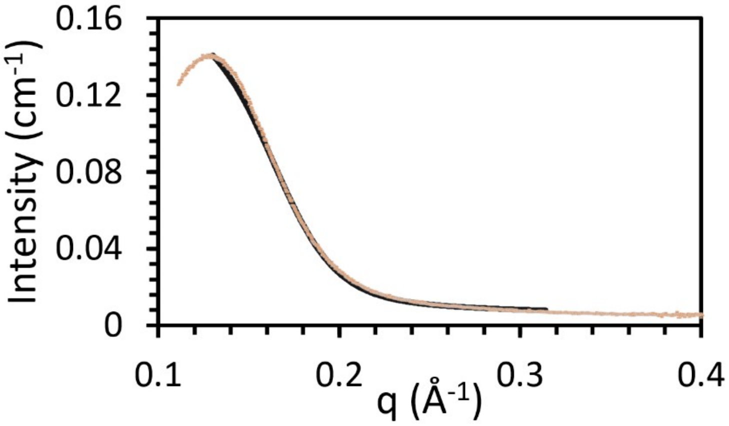

Figure S3: SAXS data of the surfactant in aqueous solution, showing raw data points (x) and the SASView fit (solid line) corresponding to a spherical particle with R_g of 16.6 Å, in good agreement with the small particles observed in samples containing modified avidin and IL.

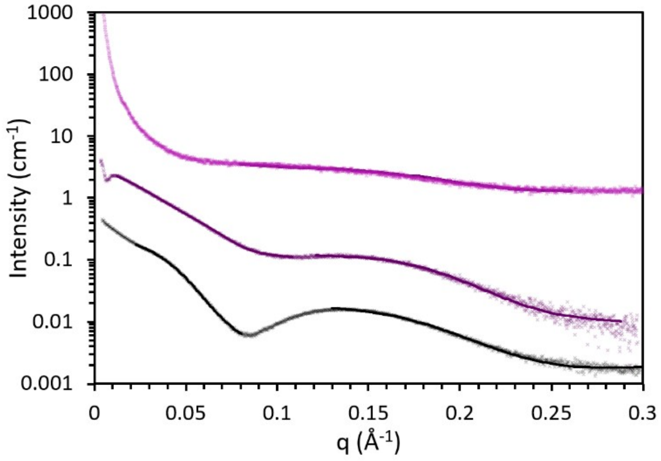

Figure S4: SAXS data for modified avidin in 100 mM phosphate buffer (black), 10 wt% [Cho][Phe] in water (dark purple) and 50 wt% [Cho][Phe] in water (light purple), showing the raw data (x) fitted to a 'stickyhardsphere' model (solid line). The three data sets are shown offset for clarity. For the 10 wt% [Cho][Phe] and buffer samples, 2 separate fits are shown for low and high q values, corresponding to the modified protein and surfactant micelle respectively, while for 50 wt% [Cho][Phe] only the high q-value (surfactant micelle) fit was possible, as the data loses definition at low q values.

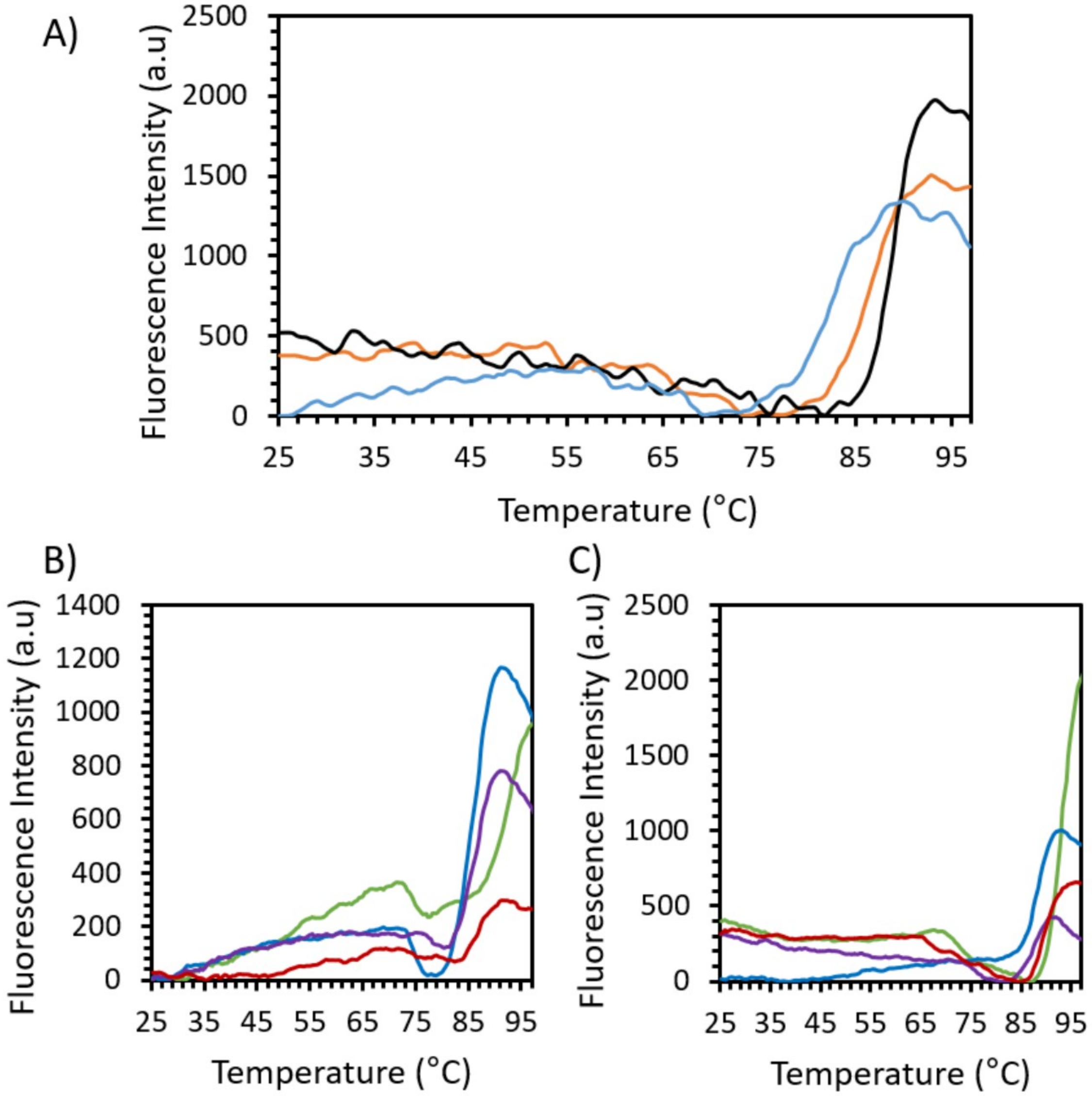

Figure S5: Fluorescence diagrams from the DSF of unmodified avidin in A) pH 5.1 citrate-phosphate buffer (orange) pH 7.1 citrate-phosphate buffer (black), pH 9.1 tris buffer (blue). B) In 10 wt% aqueous IL solutions of [Cho][Asp] (green), [Cho][Met] (blue), [Cho][Phe] (purple) and [Cho]Cl (red). C) In the same 10 wt% aqueous IL solutions but with added 0.05M, pH 7.1 citrate-phosphate buffer.

Method S1: Methodology for thermodynamic calculations from CD and DSF data

For DSF data, samples were heated from 25–99 °C at 4 °C min^{-1} and fluorescence spectra were recorded at 570 nm. For variable temperature CD data, samples were heated from 25–95 °C, and sequential CD spectra recorded at 2 °C intervals. This data was converted into a plot of the protein fraction denatured (f_D) at a given temperature by using a two-state model of denaturation, which assumes an equilibrium between the native (N) and denatured (D) states of the protein, with equilibrium constant (K_D). For the native state we can approximate a linear dependency of the fluorescence or CD signal on temperature, with

$$y_N = a_N + b_N T$$

where y_N is the (predicted) fluorescence or CD signal of native avidin at temperature T, and a_N and b_N are temperature independent variables calculated by plotting the fluorescence at 570 nm or CD signal (at 228 nm for unmodified and 216 nm for modified avidin) against temperature, which gives a roughly sigmoidal plot. For the native state, the region at low T should be approximated to a linear fit, with intercept and gradient a_N and b_N respectively, and y_N can then be calculated for all T. The equivalent linear relationship between y_D, the predicted fluorescence or CD signal of denatured avidin, and T can be derived, calculating a_D and b_D from a linear fit at high T values. Since avidin is highly thermally stable, in many cases there were insufficient points to give a linear region at high T. In these cases, a_D was approximated as the fluorescence or CD signal at the maximum temperature and b_D as 0. From here the fraction denatured could be calculated as

$$f_D = \frac{(y - y_N)}{(y_D - y_N)}$$

where y was the measured fluorescence or CD signal. This was then used to calculate the K_D and Gibbs free energy of denaturation (ΔG_D)

$$K_D = \frac{f_D}{1 - f_D}$$

$$\Delta G_D = - RT ln K_D$$

for ideal gas constant R. ΔG_D can be plotted linearly against temperature in the transition region

(-5 kJ mol^{-1} < ΔG_D < 5 kJ mol^{-1}), which describes the behaviour of ΔG_D around the denaturationtemperature (T_m) and allows calculation of T_m as the value of T for which $\Delta G_D = 0$, as well as estimation of the enthalpy (ΔH_m) and entropy (ΔS_m) of denaturation from:

$$\Delta G_D = 0 = \Delta H_m - T_m \Delta S_m$$

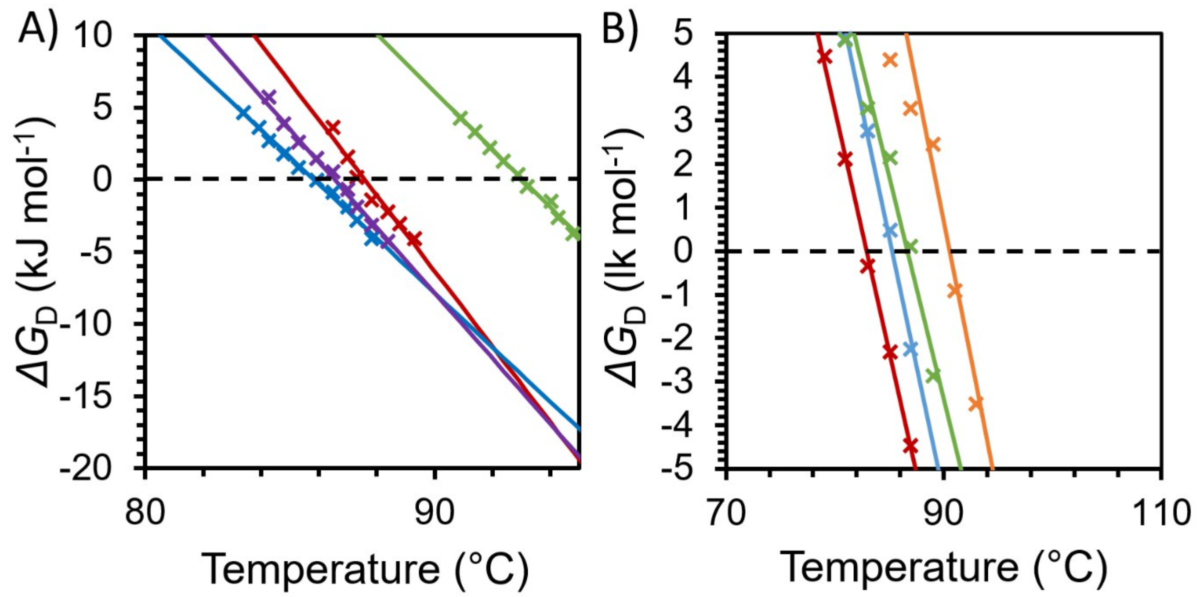

Figure S6: The free energy of denaturation ΔG_D, as calculated using the method in ESI Method S1, plotted against temperature. A) From the DSF fluorescence data and fraction denatured plot for avidin in 10 wt% [Cho][Asp] (green, 10 wt% [Cho][Met] (blue), 10 wt% [Cho][Phe] (purple) and 10 wt% [Cho]Cl (red). B) From the temperature-variable CD data and fraction denatured plot for unmodified avidin in 10 wt% [Cho][Hex] (orange), 30 wt% [Cho][Hex] (red), 30 wt% [Cho]Cl (blue) and 50 wt% [Cho]Cl (green). These plots were used to determine T_m and hence ΔH_m and ΔS_m as given in ESI Table S1. Equivalent plots were used to calculate the same thermodynamic properties for the pH buffer control solutions and the IL solutions with added citrate-phosphate buffer from DSF and modified avidin from CD.

Table S1: Thermodynamics data as determined from the raw fluorescence data and fraction denatured plots (see ESI Figures S4 for raw data, Figure S5 for processed data and Method S1 for method calculation details). – indicates no buffer added to a sample.

**Solution**	**Buffer (Conc (M))**	T_m **(°C)**	ΔH_m **(kJ mol^{-1})**	ΔS_m **(J K^{-1} mol^{-1})**	$\Delta\Delta G_D$[a] **(kJ mol^{-1})**
		Buffer Only			
pH 7.1	Cit-phos (0.05)	88.7 ± 0.0	233 ± 9	2620 ± 60	0
pH 5.1	Cit-phos (0.05)	86.4 ± 0.1	148 ± 4	1710 ± 20	-4.1 ± 0.1
pH 9.1	Tris (0.05)	82.9 ± 0.1	127 ± 9	1530 ± 60	-8.9 ± 0.7
		10 wt% IL Solutions			
[Cho]Cl	-	87.6 ± 0.1	229 ± 37	2620 ± 240	-3.0 ± 0.5
[Cho]Cl	Cit-phos (0.05)	90.4 ± 0.1	246 ± 18	2730 ± 120	4.3 ± 0.3
[Cho][Asp]	-	93.1 ± 0.1	186 ± 7	2000 ± 40	8.6 ± 0.3
[Cho][Asp]	Cit-phos (0.05)	93.2 ± 0.0	218 ± 12	2340± 70	10.5 ± 0.6
[Cho][Met]	-	85.8 ± 0.1	161 ± 8	1880 ± 50	-5.5 ± 0.3
[Cho][Met]	Cit-phos (0.05)	88.1 ± 0.0	205 ± 7	2330 ± 50	-1.5 ± 0.1
[Cho][Phe]	-	86.6 ± 0.0	196 ± 13	2270 ± 90	-5.0 ± 0.3
[Cho][Phe]	Cit-phos (0.05)	87.5 ± 0.1	233 ± 10	2670 ± 60	-3.2 ± 0.1

Table S2: The wavelength at which the Chirascan CD reached maximum HV on a decreasing scan from 260 – 190 nm, for each IL at the maximum wt% given and at 15 wt%. When the HV of the detector approaches maximum the signal can no longer be optimised by the machine, so results become less reliable. Higher HV values also increase noise. HV should not exceed 80% of its maximum near the wavelength(s) of interest.

**IL**	**Maximum concentration tested (wt%)**	**Wavelength of max HV at max conc. (nm)**	**Wavelength max HV at 15 wt% (nm)**	**Suitable for analysis by CD**
[Cho][Hex]	80	232.5	217.5	Yes
[Cho][Ger]	50	260.0	260.0	No
[Cho][Asp]	70	232.0	220.5	No
[Cho][Met]	80	260.0	260.0	No
[Cho][Phe]	15	240.5	240.5	No

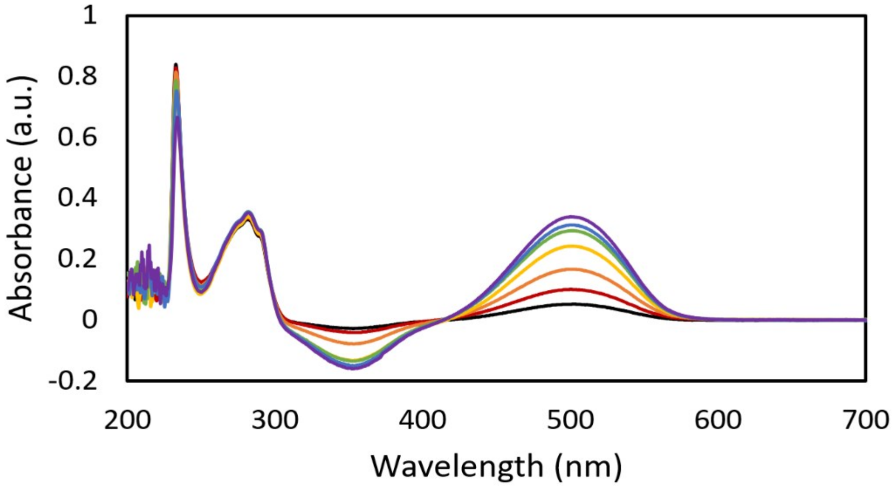

Figure S7: UV-Vis data from the sequential HABA-avidin binding, showing the growth of the positive peak at 500 nm representing the bound HABA in the protein sample and the negative peak at 350 nm, which occurred due to the background subtraction of a protein free sample containing an equal amount of HABA, but all unbound, from the avidin containing sample. The HABA:avidin ratio in each sample was 1 (black), 2 (red), 4 (orange), 8 (yellow), 12 (green), 16 (blue) and 23 (purple).
